# Intensified tuberculosis and HIV surveillance in a prison in Northeast India: Implementation research

**DOI:** 10.1371/journal.pone.0219988

**Published:** 2019-07-29

**Authors:** Tarun Bhatnagar, Malsawmtluangi Ralte, Lalhriatzuali Ralte, Chawnglungmuana, L. Sundaramoorthy, Lily Chhakchhuak

**Affiliations:** 1 ICMR School of Public Health, ICMR-National Institute of Epidemiology, Chennai, India; 2 Mizoram State AIDS Control Society, Ministry of Health and Family Welfare, Aizawl, India; 3 Department of Health and Family Welfare, Mizoram, Aizawl, India; 4 SHALOM NGO, Aizawl, India; University of Ghana College of Health Sciences, GHANA

## Abstract

Structural and individual level factors in prisons create challenges towards detection and management of HIV/tuberculosis. WHO and India’s HIV/tuberculosis control programs recommend intensified case finding in prisons. Low HIV and tuberculosis detection rates suggest poor implementation of existing surveillance strategies within the prison healthcare system in Mizoram’s capital city of Aizawl. We explored the operational feasibility of implementing the intensified case finding strategy in Aizawl central prison. We implemented the intensified screening through entry screening of new inmates, mass screening of resident inmates and exit screening at release. We set up digital chest radiography, sputum smear microscopy and HIV testing facilities within the prison and referral to external facility for Cartridge Based Nucleic Acid Amplification Test (CBNAAT). We screened 738 inmates (Male: 626; Female: 112). Of 53% inmates having presumptive tuberculosis symptoms, 37% underwent sputum microscopy. We detected 14 new tuberculosis cases; overall tuberculosis positivity 1.9%. We tested 65% of 657 inmates for HIV, of which 41 new cases were detected; overall HIV positivity 16.5%. Three male inmates had HIV-tuberculosis co-infection. It is feasible to implement intensified case detection for tuberculosis/HIV in the prison with inter-departmental coordination, albeit with certain challenges.

## Introduction

The Global Plan to End TB 2016–2020 provides strategies to preventing TB, active case finding and contact tracing, including work in varied epidemic and socioeconomic environments with the target of reaching at least 90% of the most vulnerable, underserved, at-risk populations [1]. Prisoners are an especially vulnerable population, often coming from the lowest socioeconomic groups in societies, minority or migrant groups, with increased risk of ill health, high levels of mental disorders, risk of self-harm, and a higher incidence of tuberculosis, multi-drug resistant tuberculosis and human immunodeficiency virus (HIV) compared to the general population [2–3]. The incidence of tuberculosis in prisons is reported to be 5–70 times higher than within the general population [4–7]. Overcrowding, inadequate ventilation, and lack of quarantine facilities promote efficient transmission of tuberculosis [6–8]. This is further exacerbated by individual level factors, including concomitant HIV infection, poor nutrition and hygiene, drug addiction, needle sharing and unsafe sex [9–11]. Delayed case detection, poor contact detection, inadequate treatment, high turnover of prisoners, and poor implementation of infection control measures hamper tuberculosis control in prisons [5,12]. Structural issues such as lack of training in standard tuberculosis treatment and care practices, insufficient laboratory capacity and diagnostic tools, interrupted supply of medicines, weak integration between civilian and prison medical services, and low policy/funding priority for prison healthcare create additional challenges [7,11,13].

Prisons in India are heavily crowded with majority of the inmates being uneducated, poor, and belonging to marginalized or socially disadvantaged groups [14]. Tuberculosis and HIV are commonly reported among the prison inmates in India [15–18]. Although tuberculosis and sexually transmitted infections are treated by the prison medical doctors, in some prisons in India, HIV-positive prisoners are lodged in the same barrack with those suffering from tuberculosis [19]. Informal power structures among prisoners, the barter system, and a long route to access health care service contribute to the poor health care in prisons. Reports suggest that Indian prisons are not able to provide the basic living standards in terms of ventilation, sanitation and hygiene as prescribed in the model prison manual for India and even the primary health care services being provided are of poor quality [20,21].

Mizoram reported the country’s highest adult HIV prevalence of 2.04% [22]. Central Jail in Mizoram State is located in the capital city of Aizawl, with 40% inmates being injecting drug users. As per prison records of nearly 800 inmates, since 2012 only 14 cases of tuberculosis were detected and put on treatment. Only 14% got voluntarily tested for HIV, of which 9.8% and 14% were reactive in 2014 and 2015, respectively [Personal communication]. The current scenario in Central Jail is suggestive of suboptimal case detection, poor referral and linkages between HIV and tuberculosis services, and inadequate follow-up services.

Routine opt-out HIV testing in prisons done in a voluntary, informed and non-coercive manner can increase case detection, particularly in high HIV prevalence settings. It can help to provide linkage to HIV treatment and care, thus preventing transmission within prisons and in the community [23–30]. World Health Organisation (WHO) recommends setting up of active case finding system for tuberculosis in prisons including developing linkages with health services outside prison [31]. The WHO and International Union Against Tuberculosis and Lung Disease have urged to prioritize tuberculosis prevention and control in prison settings including building evidence through operational research [32, 33]. Our objectives were to examine the feasibility and performance of intensified case finding strategy for tuberculosis/HIV case detection among inmates of Central Jail, Aizawl.

## Materials and methods

### Study setting

Central Jail, Aizawl is the largest prison in Mizoram state. The prison capacity is 545 inmates, including 456 males and 89 females. The prison had provision for a medical inspection room, with a doctor, three nurses, pharmacist, laboratory technician, three male inmates designated as medical assistants, and one female inmate designated as laboratory assistant. Inmates who voluntarily sought care were managed based on the presenting signs and symptoms. There were no facilities for sputum examination and radiography. The prison doctor referred all tuberculosis suspects either to the Designated Microscopy Centres (DMC) at Kulikawn Tuberculosis Unit, 10 kilometres from the prison, or to the District Tuberculosis Centre (DTC) at State Referral Hospital, Falkawn, 30 kilometres from the prison, for diagnosis and follow-up. There was no separate ward in the prison to isolate tuberculosis patients. Supply of anti-tuberculosis drugs was inconsistent. Mizoram State AIDS Control Society provided the facility for routine HIV screening inside the prison. HIV counseling and testing was provided through mobile unit of integrated counseling and testing centre (ICTC) that visited the Central Jail once in three months. Inmates were referred to Civil Hospital, Aizawl for HIV confirmatory test and antiretroviral treatment (ART). There was provision for one ambulance and on any given day only two inmates could be transported at a time to government health institutions in Aizawl city.

### Implementation strategy

We did ‘entry screening’ of new inmates, ‘mass screening’ of inmates residing within the prison (serving their sentence before the beginning of the study), and ‘exit screening’ of those released from the prison without mass screening during April-July 2017. Intensified screening, done over two days, included a clinical examination by the prison medical officer, tuberculosis and HIV risk assessment by the counselors, tuberculosis symptom screening (cough>2 weeks, fever>2 weeks, significant weight loss, hemoptysis and night sweat) and collection of two sputum samples (spot and early morning) from presumptive tuberculosis cases by the nurse, chest radiography of presumptive tuberculosis cases by x-ray technician and opt-out HIV testing by the laboratory technician.

Inmates reactive to the first HIV test were confirmed when the mobile ICTC counselor and laboratory technician visited the Central Jail once every week during the study period. HIV screening was done using Meriscreen HIV1/2 WB (whole blood) testing kit. Tests showing reactive results were then confirmed using Comb AIDS RS Advantage HIV-1 and Signal Flow HIV-1 testing kits. The tests were based on the development of color bands within 20 minutes of adding 20 microlitres of blood and the diluents to interpret the results as non-reactive or reactive for HIV 1/2 antibodies. The test was considered as invalid if no band appeared on Control Line or the band appeared only on Test Line.

Sputum samples were taken for smear microscopy to DTC, Falkawn by the project staff. The smear was prepared using the yellow purulent portion of the sputum with stepwise addition of 1% carbol fuchsin, 25% sulphuric acid and 0.1% methylene blue as per standard operating procedure. Project staff collected the results from DTC after two-three days.

Sputum samples of HIV positive inmates, collected in falcon tubes, were sent for CBNAAT (*Cepheid*) to DTC, Falkawn. The technician mixed 2–5 ml sputum sample with 8 ml sample reagent followed by incubation at room temperature and loading of the cartridge with 2 ml mixed sample into the genexpert. Four samples could be analysed at one time. It took two hours to complete one cycle. The test was performed for the second time only if the test results were “invalid” or “Rif Indeterminate”. The test was repeated on the same sample after trouble shooting (as per the user manual) in case of “errors” or “no results”. Results were collected by the project staff after one week.

Digital radiography machine was used for chest radiographs. Two views of the chest were taken, one by positioning the inmate in a way that the chest was pressed against the image plate with hands on hips and for the second view, the inmate’s side was pressed against the image plate with arms elevated. Inmates who were unable to stand were made to lie down on the table. The inmates were instructed to not move until the procedure was complete in order to minimize the chances of blurring. Results were recorded on a detector and were displayed in a digital format on a computer screen. The same were printed on radiograph films and sent to the radiologist for reading and reporting twice a week. The reports were collected after two-three days by the project staff. Inmates with radiographical signs suggestive of tuberculosis, such as mediastinal/hilar lymphadenopathy, consolidation seen as opacity in a segmental or lobar distribution, cavitation, pleural effusion, segmental or lobar atelectasis, lobar hyperinflation, mucoid impaction, postobstructive pneumonia, and/or randomly distributed diffuse nodules, were examined by the prison doctor and referred to DTC for initiating treatment.

Review of tuberculosis risk factors and symptoms, HIV test results, interpretation of chest radiography, sputum smear microscopy and Cartridge Based Nucleic Acid Amplification Test (CBNAAT) results were completed in 4–5 days. Testing was done as per the tuberculosis diagnostic algorithm of India’s tuberculosis control program and HIV testing guidelines of National AIDS Control Organisation (NACO) [34, 35]. Inmates detected HIV positive were counselled by the mobile ICTC counsellor at the prison site. Their blood samples were sent to Civil Hospital, Aizawl for investigations prior to initiating ART and results were informed on the same day to the prison doctor. HIV positive inmates were referred to the Civil Hospital, Aizawl for further counselling, registration and initiation of ART.

### Data collection

Trained investigators used structured questionnaire in Mizo language to collect information on socio-demographics and TB/HIV-related risk assessment from the inmates. Structured case forms were used to document clinical characteristics and laboratory results.

### Data management and analysis

Data entry was done using Epi-Info 7.2.2.6 generated formats. Proportions were calculated for pre-intervention socio-demographic, behavioral and clinical characteristics of prison inmates; post-intervention tuberculosis/HIV proportions, including bacteriological confirmed tuberculosis (based on sputum microscopy and/or CBNAAT), clinically diagnosed tuberculosis (based on chest radiography), HIV positive and HIV-tuberclosis coinfection. SPSS was used for data analysis.

### Ethical considerations

Participation in the study was voluntary. The inmates had the option of not getting screened. Written informed consent were obtained from all study participants. Identities of the inmates who did not participate were not revealed to the prison officials. Furthermore, the prison officials ensured that none of the inmates would be penalised in any manner in case they did not wish to participate in the screening and continued to receive all medical services as needed. The protocol was approved by Institutional Ethics Committee, Civil Hospital, Aizawl. Approvals were obtained from the Mizoram State AIDS Control Society, Department of Health & Family Welfare, Department of Hospital & Medical Education, Inspector General of Prisons and the Atomic Energy Regulatory Board.

## Results

Screening of 738 inmates, 626 (84.8%) males and 112 (15.2%) females was done. Of these, 248 (33.6%) underwent entry screening, 477 (64.6%) mass screening and 13 (1.8%) exit screening. During the study period 248 out of 475 (52%) inmates who entered the prison and 490 out of remaining 1922 inmates (25%) underwent screening. Table 1 shows the profile of 738 inmates, of whom 557 (75%) were 25–49 years old, 284 (38.5%) were divorced/separated/widowed, 237 (32.1%) had income below poverty line and 718 (97.3%) were literate. Overall, 295 (40%) had a prior history of incarceration and 472 (64%) had spent three months or less in the prison during their current term.

**Table 1:** Inmate profile, Central Jail, Aizawl, Mizoram, 2017.

Characteristics	Male (N = 626) n (%)	Female (N = 112) n (%)	Total (N = 738) n (%)
Age (years)			
18–24 25–49 50+	93 (14.9) 466 (74.4) 67 (10.7)	10 (8.9) 91 (81.3) 11(9.8)	103 (14.0) 557 (75.5) 78 (10.6)
Marital status			
Divorced/Separated/Widowed Married Unmarried	230 (36.7) 224 (35.8) 172 (27.5)	54 (48.2) 54 (48.2) 4 (3.6)	284 (38.5) 278 (37.7) 176 (23.8)
Income below poverty line	199 (31.8)	38 (33.9)	237 (32.1)
Education status			
Illiterate 1–5 class 6–8 class 9–12 class Graduate/Post graduate	13 (2.1) 112 (17.9) 196 (31.3) 269 (43.0) 36 (5.7)	7 (6.2) 27 (24.1) 30 (26.8) 47 (41.9) 1 (0.9)	20 (2.7) 139 (18.8) 226 (30.6) 316 (42.8) 37 (5.0)
Occupation prior to current prison term			
Unskilled labour Skilled labour Small/Medium business Service Unemployed Home maker Others	216 (35.0) 166 (27.0) 61 (9.7) 66 (11.0) 54 (8.6) 0 63 (10.1)	17 (15.2) 0 52 (46.4) 5 (4.5) 0 28 (25) 10 (8.9)	233 (31.6) 166 (22.5) 113 (15.3) 71 (9.6) 54 (7.3) 28 (3.8) 73 (9.9)
Prior history of incarceration	249 (39.8)	46 (41.1)	295 (40.0)
Time in prison during current term			
<1 week 1 week—3 months >3–12 months >1 year	237 (37.9) 143 (22.9) 110 (17.6) 136 (21.7)	40 (35.7) 52 (46.4) 12 (10.7) 8 (7.1)	277 (37.5) 195 (26.4) 122 (16.5) 144 (19.5)

Table 2 describes HIV-related risk behaviors among the male and female inmates. Overall, 254 (34.4%) and 20 (2.7%) inmates reported ever injecting drugs outside and inside the prison, respectively; more so among males (37.9%, 3%) than females (15.2%, 0.9%). Among those reporting injecting drug use, 162 (63.8%) and 16 (80%) reported sharing needles/syringes outside and inside the prison, respectively. Overall, 183 (24.8%) inmates reported having sex with multiple partners in the last one year, more so among males (38%) than females (7%). Only 123 (16.7%) inmates reported using condoms in last one year; similarly among males and females. Thirty two (5.1%) of 621 male inmates reported having sex with a female sex worker (FSW) in last one year, of whom 14 (43.8%) reported never using condoms with FSW.

**Table 2:** HIV risk behavior of inmates, Central Jail, Aizawl, Mizoram, 2017.

Characteristics	Male (N = 626) n (%)	Female (N = 112) n (%)	Total (N = 738) n (%)
Ever used injection drugs outside prison	237 (37.9)	17 (15.2)	254 (34.4)
Needle/syringe shared outside prison	(N = 237)	(N = 17)	(N = 254)
Always Sometimes Never	8 (3.4) 143 (60.3) 86 (36.3)	3 (17.6) 8 (47.1) 6 (35.3)	11 (4.3) 151 (59.4) 92 (36.2)
Ever used injection drugs inside prison	19 (3.0)	1 (0.9)	20 (2.7)
Needle/syringe shared inside prison	(N = 19)	(N = 1)	(N = 20)
Always Sometimes Never	9 (47.4) 6 (31.6) 4 (21.1)	1 (100.0) 0 (0.0) 0 (0.0)	10 (50.0) 6 (30.0) 4 (20.0)
Sex with multiple partners in last one year			
Regularly Occasionally Never	34 (5.4) 141 (22.5) 451 (72.0)	1 (0.9) 7 (6.2) 104 (92.9)	35 (4.7) 148 (20.1) 555 (75.2)
Condom use in last one year			
Every time Sometimes Never	27 (4.3) 79 (12.6) 520 (83.1)	3 (2.7) 14 (12.5) 95 (84.8)	30 (4.1) 93 (12.6) 615 (83.3)
Sex with FSW* in last one year	(N = 621)		
Regularly Occasional Never	8 (1.3) 24 (3.8) 589 (94.1)		
Condom use with FSW* in last one year	(N = 32)		
Every time Sometimes Never	7 (21.9) 11 (34.4) 14 (43.8)		
Ever got tattoo outside prison	192 (30.7)	11 (9.8)	203 (27.5)
Ever got tattoo inside prison	117 (18.7)	0 (0.0)	117 (15.9)
Ever had blood transfusion	58 (9.3)	19 (17.0)	77 (10.4)
Ever shared razor blade outside prison	49 (7.8)	4 (3.6)	53 (7.2)
Ever shared razor blade inside prison	56 (8.9)	2 (1.8)	58 (7.9)

* FSW: Female sex worker

As shown in Table 3, 156 (21.1%) of 738 inmates had a family member diagnosed with tuberculosis, among whom 134 (85.9%) were reported to be under treatment. Overall, 622 (84.3%) self-reported as current smokers, more so among males (89.3) than females (56.2%). Two hundred four (27.6%) inmates self-reported as current alcohol users, with males (28.6%) reporting slightly higher than females (22.3%).

**Table 3:** Tuberculosis risk factors among inmates, Central Jail, Aizawl, Mizoram, 2017.

Characteristics	Male (N = 626) n (%)	Female (N = 112) n (%)	Total (N = 738) n (%)
Family member with tuberculosis	135 (21.6)	21 (18.8)	156 (21.1)
Family member with tuberculosis on treatment	(N = 135)	(N = 21)	(N = 156)
	116 (85.9)	18 (85.7)	134 (85.9)
Smoking status			
Current smoker Past smoker Never smoker	559 (89.3) 21 (3.4) 46 (7.3)	63 (56.2) 5 (4.5) 44 (39.3)	622 (84.3) 26 (3.0) 90 (12.2)
Alcohol intake			
Current Past Never	179 (28.6) 353 (56.4) 94 (15.0)	25 (22.3) 36 (32.1) 51 (45.5)	204 (27.6) 389 (52.7) 145 (19.6)
Co-morbidity	159 (25.4)	26 (23.2)	185 (25.1)

As per Table 4, prior to the current screening 143 (19.4%) of 738 inmates had been tested for tuberculosis, 15 (2%) were positive and 610 (82.7%) were not aware of their tuberculosis status. On screening 391 (53%) inmates having at least one of the five presumptive tuberculosis symptoms were detected, more so among among males (55.4%) than females (39.3%). Among them microscopic examination of two sputum samples was done from 145 (37.1%) inmates, including 136 (39.2%) males and 9 (20.4%) females. Sputum samples of 26 (25 males) HIV positive inmates underwent CBNAAT. All 391 (100%) inmates had a chest radiograph taken. Among those tested, three new cases (all males) of bacteriological confirmed tuberculosis (1.8% of 171) and 13 cases (12 males) of clinically diagnosed tuberculosis (3.3% of 391) were detected. Overall, there were 29 (3.9%) inmates with tuberculosis, including 25 (4%) males and 4 (3.6%) females during the study period.

**Table 4:** Pre- and post-screening tuberculosis status of inmates, Central Jail, Aizawl, Mizoram, 2017.

Characterisitics	Male (N = 626) n (%)	Female (N = 112) n (%)	Total (N = 738) n (%)
**Pre-screening**			
Previously tested for tuberculosis	118 (18.8)	25 (22.4)	143 (19.4)
Tuberculosis status			
Positive Negative Don't know	12 (1.9) 93 (14.9) 521 (83.2)	3 (2.7) 20 (17.9) 89 (79.5)	15 (2.0) 113 (15.3) 610 (82.7)
**Post-screening**			
Presumptive tuberculosis symptoms			
Cough > = 2 weeks Blood in cough Fever > = 2 weeks Significant weight loss Night sweat	153 (24.4) 13 (2.1) 72 (11.5) 35 (5.6) 185 (29.6)	23 (20.5) 2 (1.8) 15 (13.4) 3 (2.7) 22 (19.6)	176 (23.8) 15 (2.0) 87 (11.8) 38 (5.1) 207 (28.0)
Presumptive tuberculosis	347 (55.4)	44 (39.3)	391 (53.0)
Sputum examination (2 samples)	(N = 347) 136 (39.2)	(N = 44) 9 (20.4)	(N = 391) 145 (37.1)
Sputum positive for tuberculosis	(N = 136) 1 (0.7)	(N = 9) 0	(N = 145) 1 (0.7)
Chest radiography done	347 (100.0)	44 (100.0)	391 (100.0)
Chest radiograph suggestive of tuberculosis	12 (3.5)	1 (2.3)	13 (3.3)
CBNAAT done	25 (7.2)	1 (2.3)	26 (6.6)
CBNAAT positive for tuberculosis	(N = 25) 2 (8.0)	(N = 1) 0	(N = 26) 2 (7.7)
Detected tuberculosis positive (bacteriological/clinical)	13 (2.1)	1 (0.9)	14 (1.9)

Out of 14 (13 males, 1 female) newly detected tuberculosis positive inmates referred to DTC, Falkawn, six males started anti-tuberculosis treatment, one male was released from prison before initiation of ATT and outcome of 7 (6 males, 1 female) inmates was not known.

As per Table 5, prior to the current screening, 536 (72.6%) of 738 inmates reported to have been tested for HIV, 81 (11%) were HIV positive and 228 (30.9%) did not know about their HIV status. Of the 78 HIV positive inmates, 48 (61.5%) were currently on pre-ART or ART. Of the 657 inmates (565 males, 92 females) with negative or unknown HIV status 452 (68.8%) were tested for HIV, including 70.8% males and 56.5% females. Forty one (9.5%) of 431 inmates (10% males, 5.9% females) were detected as HIV positive. Overall, 122 (16.5%) inmates, including 99 (15.8%) males and 23 (20.5%) females, were HIV positive during the study period.

**Table 5:** Pre- and post-screening HIV status of inmates, Central Jail, Aizawl, Mizoram, 2017.

Characteristics	Male (N = 626) n (%)	Female (N = 112) n (%)	Total (N = 738) n (%)
**Pre-screening**			
Previously tested for HIV	439 (70.1)	97 (86.6)	536 (72.6)
HIV status			
Positive Negative Don't know	61 (9.7) 352 (56.2) 213 (34.0)	20 (17.9) 77 (68.8) 15 (13.4)	81 (11.0) 429 (58.1) 228 (30.9)
Known HIV+ on Pre-ART/ART	(N = 58) 22 (37.9)	(N = 20) 16 (80.0)	(N = 78) 48 (61.5)
**Post-screening**			
Tested for HIV	(N = 565) 400 (70.8)	(N = 92) 52 (56.5)	(N = 657) 452 (68.8)
Detected HIV positive	(N = 380) 38 (10.0)	(N = 51) 3 (5.9)	(N = 431) 41 (9.5)

Out of 41 (38 male, 3 female) newly detected HIV positive inmates referred to ART Centre, Civil Hospital, Aizawl, 14 (11 male, 3 female) initiated ART, eight males were registered at ART Centre and not initiated on ART, 10 males were released from prison before initiation of ART and outcome of 9 male inmates was not known.

## Discussion

The intensified screening strategy in Central Jail, Aizawl yielded 14 new tuberculosis cases with overall tuberculosis positivity of 3.9%, including known positives. This is much higher compared to the estimated prevalence of 4 per 1000 in three prisons in Karnataka and 2.11 per 1000 in the Indian general population [15]. Two-thirds of the eligible inmates were tested for HIV, of which 41 new cases were detected with an overall HIV positivity of 16.5%, including known positives, much higher than 10% prevalence among PWID in Aizawl^.^

Active involvement of key stakeholders including the prison officials, State Tuberculosis and State AIDS Control Societies and SHALOM, a local NGO was critical in effective implementation of the interventions. The prison authorities and staff were sensitized to the implementation process, including the need to maintain confidentiality. Project staff were trained in ethical issues and data collection tools and techniques. Experienced and qualified counselors provided HIV counseling to the inmates in a private one-on-one setting. Inmates had the chance to opt-out of HIV testing, or to choose not to receive the results, if they did test. It was feasible to augment the healthcare infrastructure, laboratory services for tuberculosis and HIV and human resources in the prison by mobilising resources from other departments as well. This made it possible to recognise and overcome many of the referral, linkage and security-related barriers for diagnosis, management and follow-up of the inmates. Our current intervention provided an opportunity to initiate several new practices within the prison system. Inmates with tuberculosis were isolated in a separate ward, HIV positive inmates were able to complete their blood tests prior to being referred to the nearest ART Centre for counselling, registration and initiation of ART. The time for visit to the ART centre was thus reduced from three to one day.

Implementing the intensified screening mechanisms within the prison healthcare system was challenging. Our screening coverage was low during the study period due to multiple reasons, including heavy rains, high inmate turnover, security protocols, frequent electricity outages, and administrative procedures in the prison facility. Installation of the x-ray machine was delayed due to renovation of the room as per regulatory guidelines. Some of the inmates refused to get tested, possibly due to fear of stigma and discrimination. Although all inmates were screened for presumptive tunerculosis symptoms less than half of the inmates with presumptive tuberculosis provided sputum samples for tuberculosis testing. This was despite our efforts to provide basic understanding of tuberculosis disease and importance of screening to the inmates before the beginning of the study. They were not probed further for failure to provide sputum samples considering the voluntary nature of the study. This limited our ability to estimate the prevalence of tuberculosis amog the inmates. It would be helpful in the future to understand the factors influencing this poor response in order to make the intensive screening more effective in detecting and managing new cases of tuberculosis among prison inmates. There is a need to consider mutiple strategies to engage the prison inmates in the screening process without being perceived as coercive. Follow-up of inmates after they were released from the prison was also challenging. It may be imperative to establish linkages with hospitals outside of the prison system to ensure appropriate management of tuberculosis and HIV.

The latest government guidelines for prison management propose to shift the administrative responsibility of provision of medical services from the prison department working under the Ministry of Home Affairs to the State Medical Services and Health Department that oversees the routine healthcare system [36]. This provides an opportunity to strengthen the existing prison healthcare system. Further, the guidelines for intensified surveillance of tuberculosis and HIV, including provision of laboratory services and medicines within the prison setting, isolation of tuberculosis suspects and designing tuberculosis and HIV-related information campaigns specific to the prison setting would have to be incorporated in the state prison manuals. It was operationally feasible to implement intensified screening for tuberculosis and HIV within the prison setting, albeit with certain challenges. Long term sustainability of these interventions in Aizawl and scalability to other prisons in the country remains to be evaluated.
